# Synthesis of a Coumarin-Based PPARγ Fluorescence Probe for Competitive Binding Assay

**DOI:** 10.3390/ijms22084034

**Published:** 2021-04-14

**Authors:** Chisato Yoshikawa, Hiroaki Ishida, Nami Ohashi, Toshimasa Itoh

**Affiliations:** Laboratory of Drug Design and Medicinal Chemistry, Showa Pharmaceutical University, 3-3165 Higashi-Tamagawagakuen, Machida, Tokyo 194-8543, Japan; d1702@g.shoyaku.ac.jp (C.Y.); ishida@ac.shoyaku.ac.jp (H.I.); ohashi@ac.shoyaku.ac.jp (N.O.)

**Keywords:** PPARγ ligand, coumarin, fluorescent ligand, screening, crystal structure

## Abstract

Peroxisome proliferator-activated receptor γ (PPARγ) is a molecular target of metabolic syndrome and inflammatory disease. PPARγ is an important nuclear receptor and numerous PPARγ ligands were developed to date; thus, efficient assay methods are important. Here, we investigated the incorporation of 7-diethylamino coumarin into the PPARγ agonist rosiglitazone and used the compound in a binding assay for PPARγ. PPARγ-ligand-incorporated 7-methoxycoumarin, **1**, showed weak fluorescence intensity in a previous report. We synthesized PPARγ-ligand-incorporating coumarin, **2**, in this report, and it enhanced the fluorescence intensity. The PPARγ ligand **2** maintained the rosiglitazone activity. The obtained partial agonist **6** appeared to act through a novel mechanism. The fluorescence intensity of **2** and **6** increased by binding to the ligand binding domain (LBD) of PPARγ and the affinity of reported PPARγ ligands were evaluated using the probe.

## 1. Introduction

**Figure 1:**
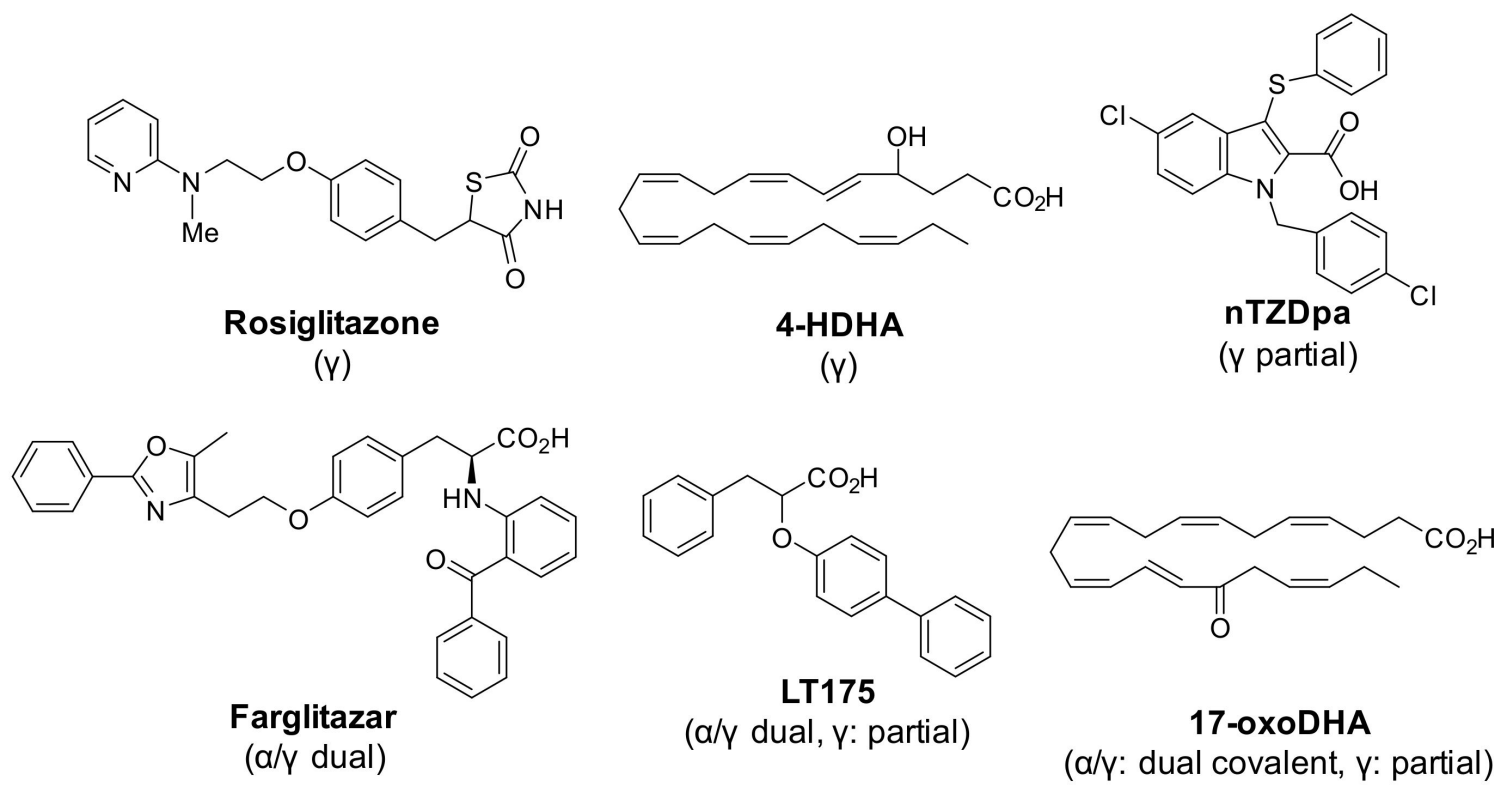
Structures of PPARγ agonists rosiglitazone and 4-HDHA, partial agonist nTZDpa, PPAR α/γ dual agonist farglitazar, dual and partial agonist LT175, and dual covalent and partial agonist 17-oxoDHA.

Peroxisome proliferator-activated receptors (PPARs) belong to the nuclear receptor superfamily and are categorized into three subtypes—PPARα, β/δ, and γ [1,2,3]. PPARγ is an important target molecule for the inflammatory disease and metabolic syndrome. PPARγ agonists were developed as antidiabetic drugs, such as rosiglitazone and pioglitazone [4] but cause adverse effects, such as heart failure, edema, and increased risk of myocardial infarction [2]. PPARγ ligands were thus developed using various strategies and include partial PPARγ agonists [5], selective PPARγ modulators, PPAR α/γ dual agonists, oxidized fatty acid agonists, antagonists, and covalent ligands [6,7,8,9,10,11,12] (Figure 1).

PPARγ was identified as a novel target of the nonsteroidal anti-inflammatory drugs (NSAIDs) action by direct binding. Like PPAR agonists, NSAIDs are believed to be fatty acid analogues and can suppress the expression of proinflammatory genes via PPARγ activation [13]. A PPARγ ligand inhibited monocyte elaboration of inflammatory cytokines and chemokine expression, and prevented microglial activation [14,15]. A fatty acid-based agonist, 4-HDHA, alleviates the symptoms of DSS-induced colitis [16]. Thus, PPARγ is a possible target molecule for anti-inflammatory as well as metabolic syndrome. The ligand binding cavity of PPARγ is versatile [17,18], allowing the design of a large number of unique compounds.

Nuclear receptor ligands, including PPARγ ligands, are often evaluated by investigating their gene transcription activity [19]. This assay is useful in identifying agonists with strong efficacy, but this is likely to overlook antagonists and partial agonists because of its weak efficacy. Therefore, the binding assay is important for exploring novel ligands. Conventional PPAR binding assays often use competition with a radioligand [20] and such assays provide superior sensitivity, but they are costly, are possible health hazards, and require laborious experimental procedures and special facilities. Fluorescent probes overcome these drawbacks [21]. A fluorescent probe for a PPAR α/γ fluorescent polarization (FP) binding assay, based on the large molecule fluorescein was developed [22,23]. In contrast, coumarins are small, and have a solvatochromic fluorescence property that increases fluorescence intensity in hydrophobic environments and decreases it in hydrophilic environments [24]. Compounds containing coumarin were developed to detect ligand binding to target proteins [25].

The coumarin skeleton is used in various fields [26,27,28] and many synthetic strategies were reported [29,30,31,32,33,34,35], including C–C bond formation reactions that require advanced knowledge and techniques for probe synthesis. In contrast, we recently reported strategies for constructing a coumarin skeleton on a target protein (TCC probe) using small molecules (coumarin precursors) [36,37]. The advantages of using a coumarin precursor in organic synthesis include incorporation of the coumarin skeleton into the ligand in the final step of synthesis in one step, and this incorporation is facile if the precursor of the ligand has a nucleophilic functional group.

**Scheme 1:**
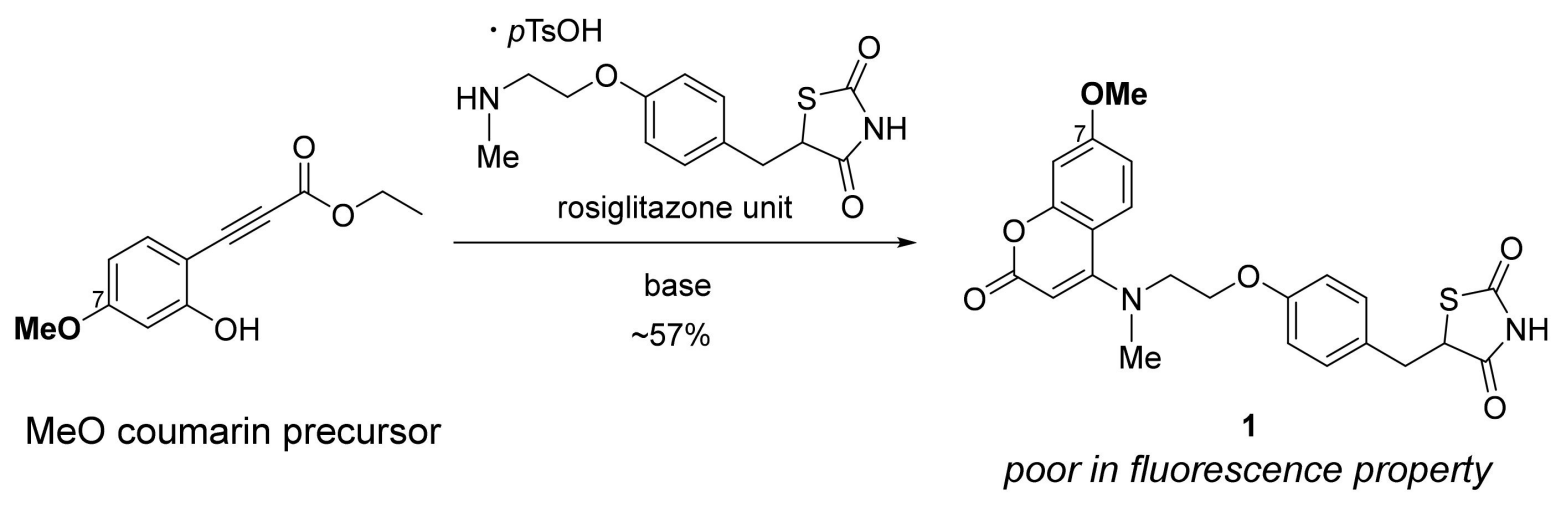
Synthesis of 7-methoxy coumarin-incorporated rosiglitazone.

We previously demonstrated the usefulness of precursors by synthesizing compound **1**, whose structure incorporates 7-methoxy (7-MeO) coumarin into the rosiglitazone (Scheme 1) [36]. However, the fluorescence property of compound **1** is poor.

Here, we synthesized a rosiglitazone-based fluorescence probe using a coumarin precursor. PPARγ binding assay shows that the probe binds to rosiglitazone. We utilized the properties of solvatochromic fluorophores instead of FP to facilitate the evaluation of the ligands.

## 2. Results

### 2.1. Design and Synthesis of a Coumarin-Based PPARγ Ligand

**Figure 2:**
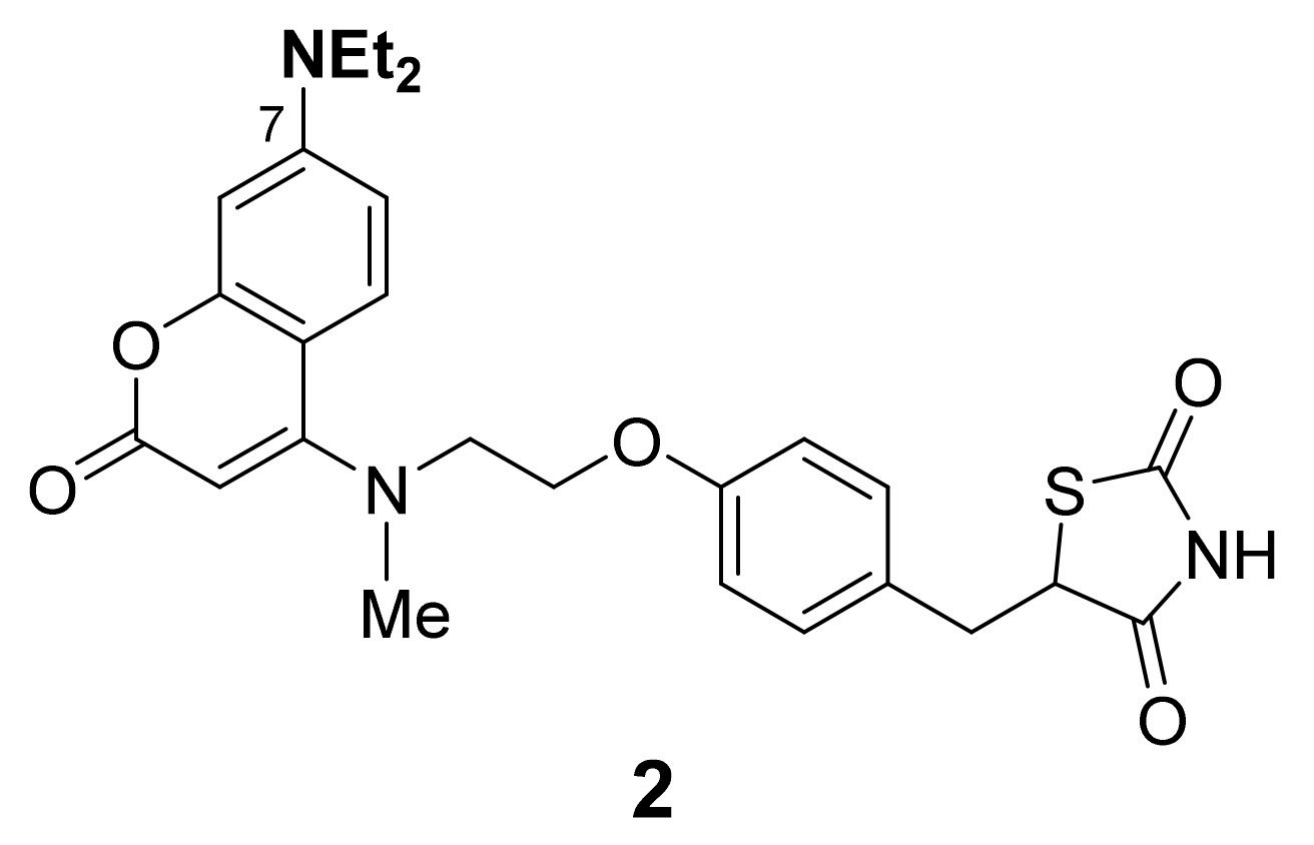
Structure of PPARγ ligand incorporated 7-diethylamino (7-Et_2_N) coumarin.

**Scheme 2:**
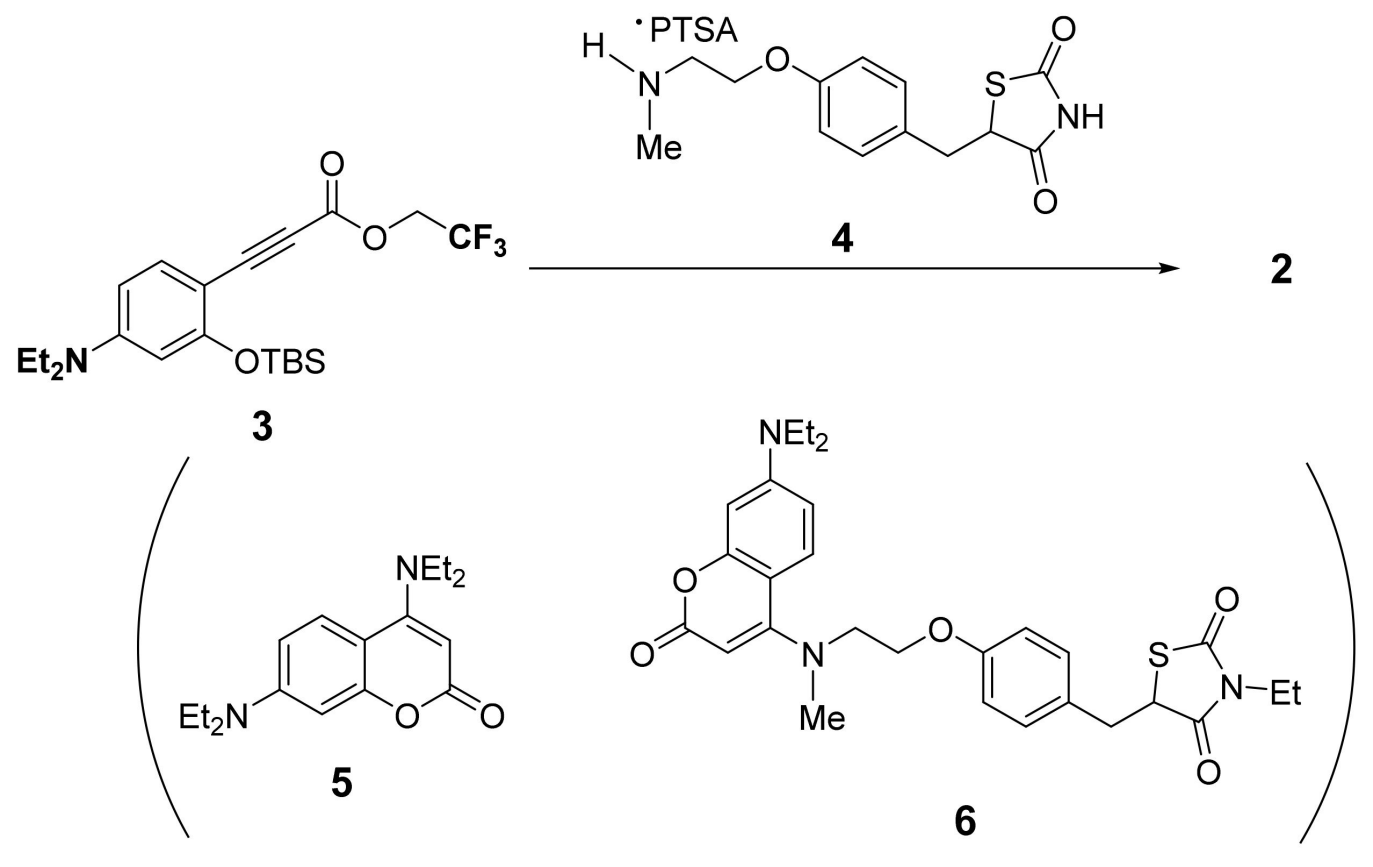
Synthesis of PPARγ ligand.

We previously synthesized the rosiglitazone-based model compound **1**, in which the terminal pyridine was replaced by the coumarin skeleton, using a coumarin precursor (Scheme 1). Coumarin in compound **1** was poor in fluorescence property. We, thus, designed compound **2**, which contained a 7-diethylamino (7-Et_2_N) coumarin unit (Figure 2). The coumarin-substituted electron-donating groups at position 7 showed strong fluorescence [38] and thus we expected the designed probe **2** to be sufficiently fluorescent to be useful for PPARγ binding assays. The precursor used was the TBS-protected form **3**, because the Et_2_N moiety destabilized the precursor. Since the Et_2_N and TBS groups were electron-donating, the reactivity (electro-deficiency) of the alkynone moiety was weak [39]. We, thus, used precursor **3** (Scheme 2) in which CH_2_CF_3_ provided an electron-withdrawing group at the ester group of the 7-Et_2_N coumarin precursor (Scheme 2). When we used Et_3_N as a base and solvent, no desired product **2** was isolated and unexpectedly, we obtained compound **5**, which was conjugated to an Et_2_N group (Scheme 2; Table 1, entry 1). We, therefore, changed the synthesis conditions from those used to synthesize 7-MeO coumarin incorporating rosiglitazone **1**. The use of DMF and Et_3_N as a solvent, gave the desired **2** in 14% yield and **5** was increased to 51% yield. Interestingly, ethylation at the thiazolidinedione (TZD) occurred to give **6** (14%) (entry 2). Yamaguchi et al. reported the conjugate addition of an ynone-containing azulene with a tertiary amine [40]. We considered that products **5** and **6** resulted from a similar mechanism (Schemes S1 and S2) and thus we reduced the amount of Et_3_N. When 3.0 equiv. Et_3_N in DMF was used, **2** was afforded in moderate yield (57%) (entry 3).

### 2.2. Transcriptional Activity

**Figure 3:**
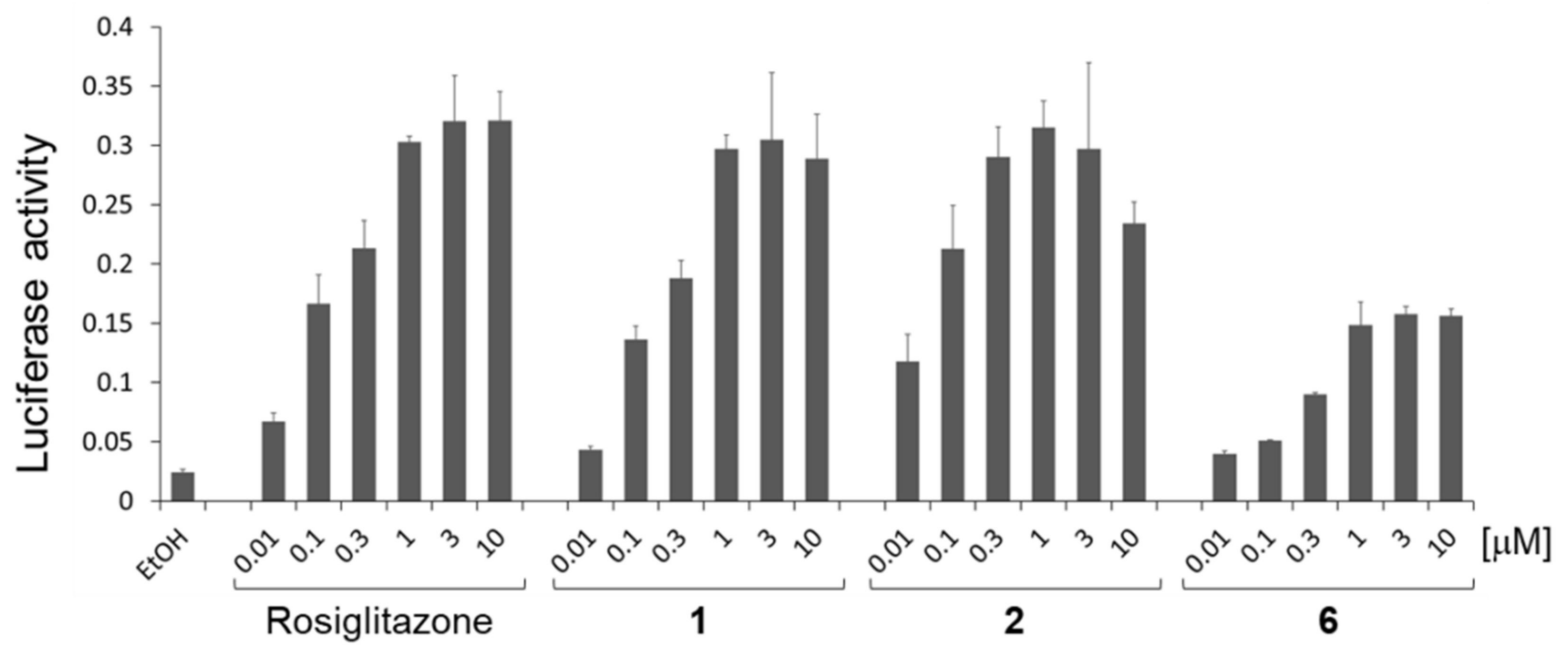
Transcriptional activity of synthetic compounds **1**, **2**, and **6** evaluated in Cos-7 cells using a dual luciferase assay with a GAL4-PPARγ chimera expression plasmid (pSG5-GALhPPARγ), a reporter plasmid (MH100×4-TK-Luc), and an internal control plasmid containing sea pansy luciferase expression constructs (pRL-CMV). The data represent the mean ± SD of three independent experiments.

The transcriptional activities of compound **1** [36], **2**, and **6** were compared for PPARγ activity, using the dual luciferase assay in Cos-7 cells (Figure 3, Table 2). Compounds **1** and **2** had activities comparable to rosiglitazone, showing that the incorporation of coumarin did not reduce ligand agonistic activity, regardless of if it was 7-MeO coumarin or 7-Et_2_N coumarin. This result suggests that incorporation of a coumarin unit into a ligand maintains biological activity because of its small structure. In contrast, compound **6** showed partial agonistic activity.

### 2.3. Fluorescence Spectra

**Figure 4:**
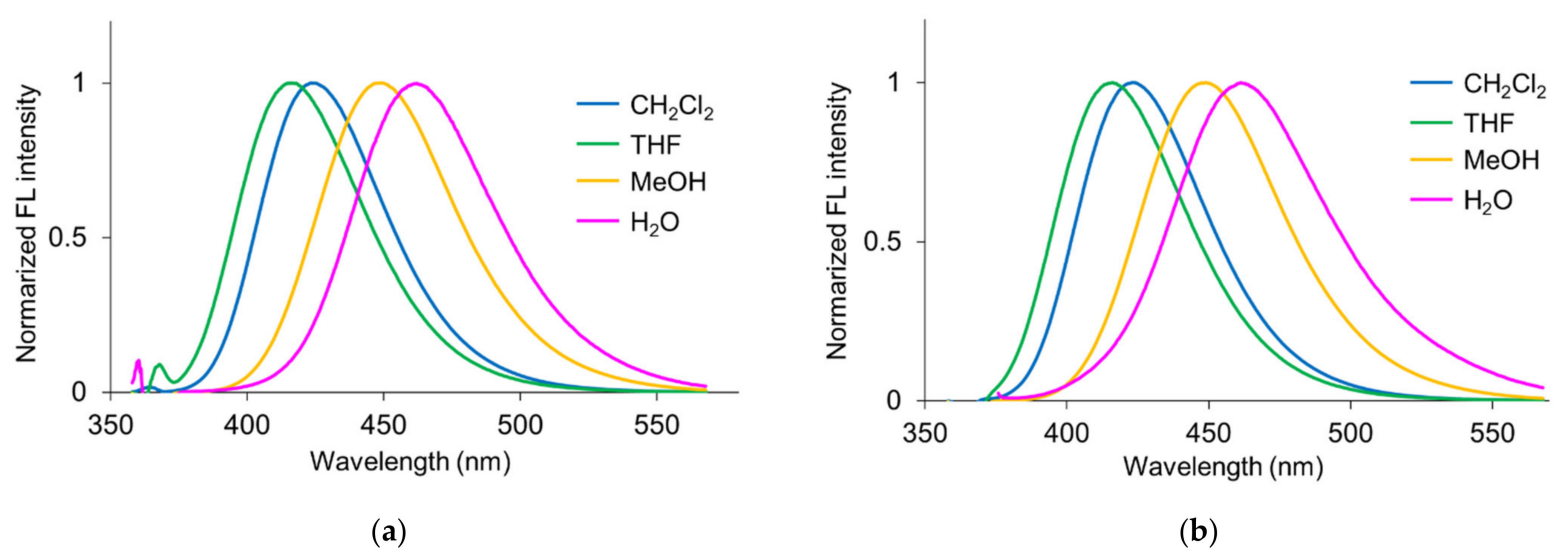
(**a**) Fluorescence spectrum of **2**, in CH_2_Cl_2_, THF, MeOH, or H_2_O (2 μM). (**b**) Fluorescence spectrum of **6**, in CH_2_Cl_2_, THF, MeOH, or H_2_O (2 μM).

We evaluated the fluorescence properties of PPARγ ligands **2** and **6** (Figure 4) by measuring the fluorescence spectra and quantum yields (Q.Y.) in CH_2_Cl_2_, THF, MeOH, or H_2_O. The Q.Y. of the 7-Et_2_N coumarins (**2**: Φ = 0.143–0.541, **6**: Φ = 0.0865–0.764) were much better than that of 7-MeO coumarin (**1**: Φ = 0.0359–0.00461) (Table 3), and exhibited a high fluorescence intensity in organic solvents and a weaker fluorescence in H_2_O. Additionally, **2** and **6** showed similar fluorescence spectral shifts, with the emission maximum shifting to a longer wavelength in polar solvent (Figure 4). The difference between Abs_max_ and Em_max_ (Δ = Em_max_ − Abs_max_) showed that the Δ value of **2** and **6** increased with increasing solvent polarity (Table 3) in the order THF < CH_2_Cl_2_ < MeOH < H_2_O. The dielectric constant of each solvent was THF: 7.4, CH_2_Cl_2_: 8.9, MeOH: 32.6, and H_2_O: 78.5 [41,42]. This correlation clarified that Et_2_N coumarins **2** and **6** exhibited a Stokes shift and thus we expected the fluorescence spectra of **2** and **6** to shift upon binding to the hydrophobic binding site of the protein.

### 2.4. X-ray Crystal Structure

**Figure 5:**
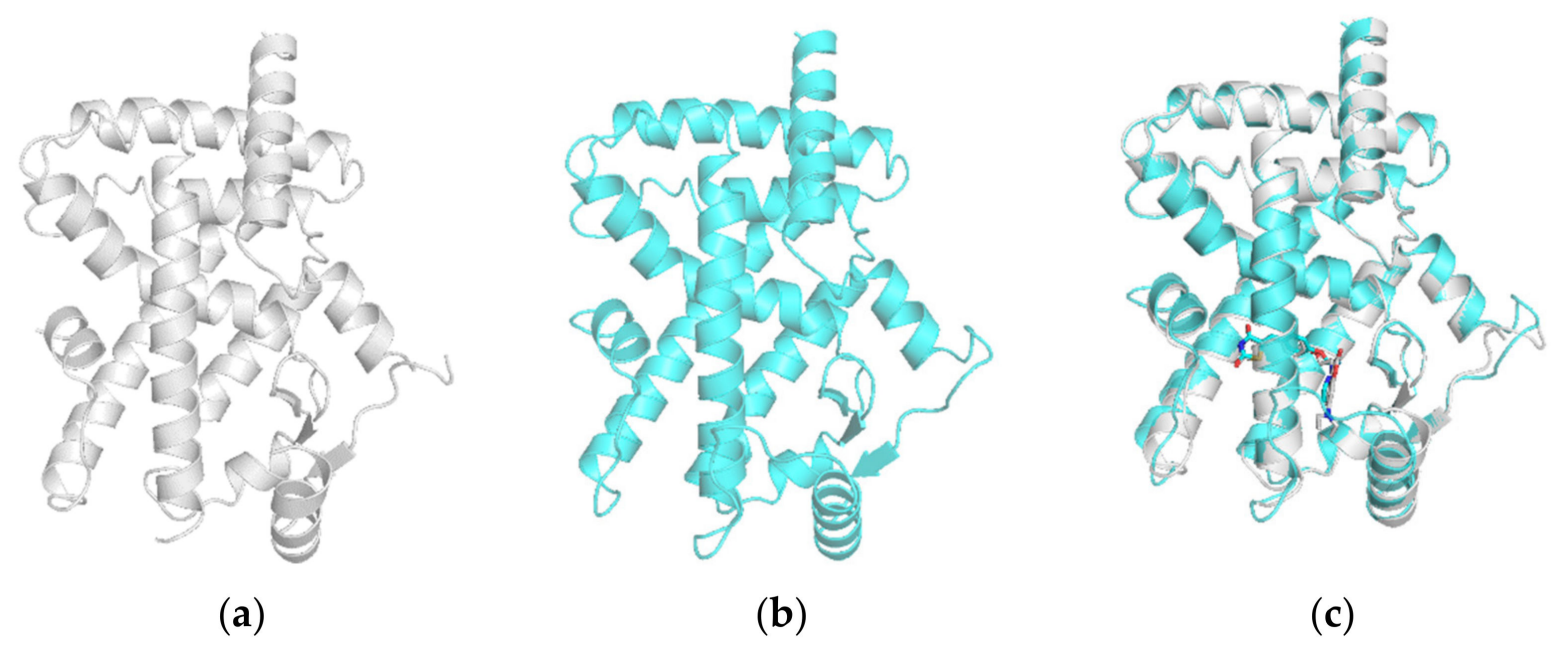
(**a**) The overall crystal structure of the **2**/PPARγ-LBD complex (PDB code: 7EFQ). (**b**) The overall crystal structure of the rosiglitazone/PPARγ-LBD complex (PDB code: 2PRG). (**c**) Comparison of the overall crystal structure of the **2**/PPARγ-LBD complex with the rosiglitazone/PPARγ-LBD complex.

**Figure 6:**
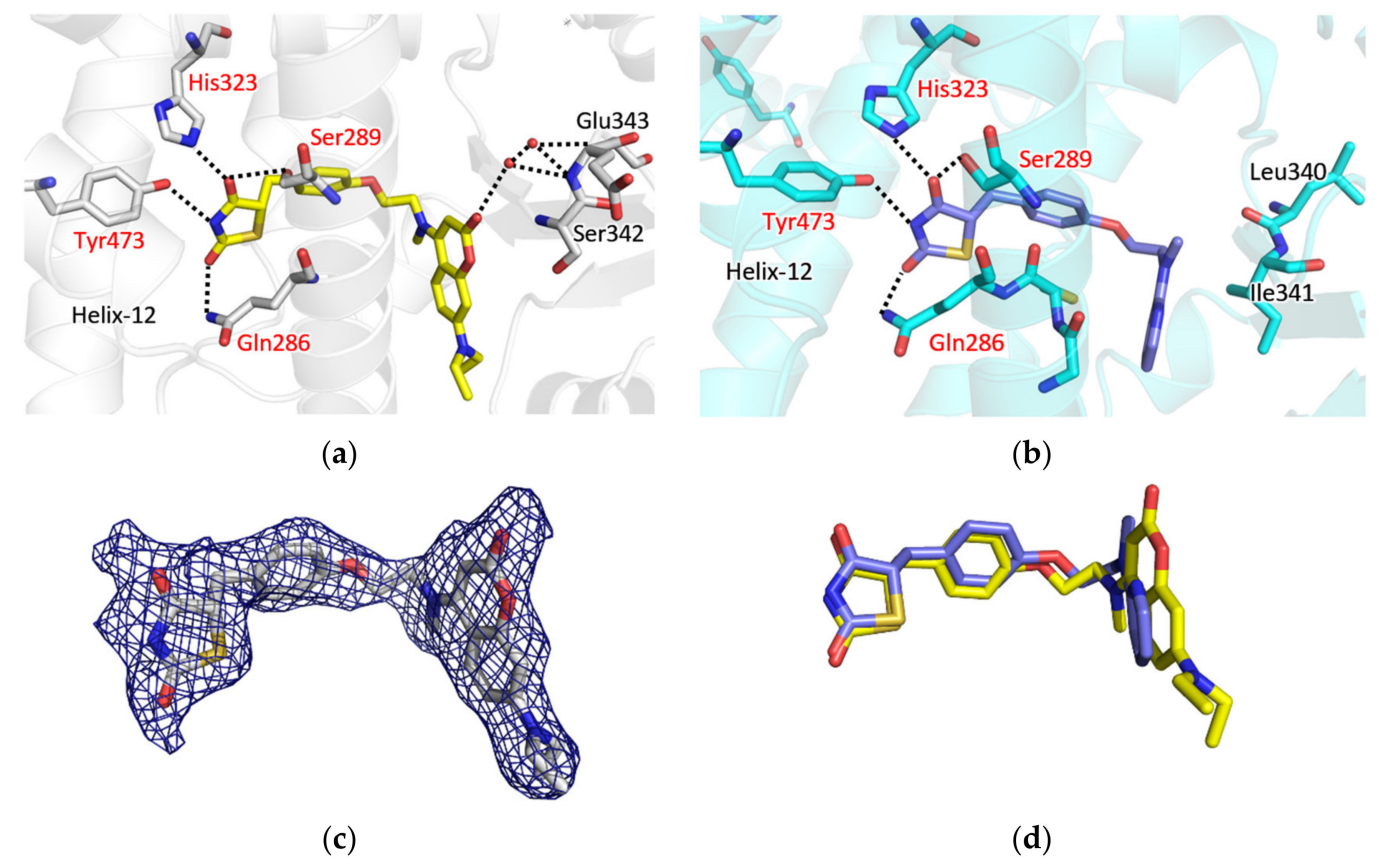
(**a**) Hydrogen bonds between **2** and hPPARγ-LBD (PDB code: 7EFQ). (**b**) Hydrogen bonds between rosiglitazone and hPPARγ-LBD (PDB code: 2PRG). (**c**) Omit map of **2** bound to hPPARγ-LBD. (**d**) Comparison of **2** with rosiglitazone bound to hPPARγ-LBD.

We attempted to crystallize human PPARγ-LBD complexed with **2** or **6** to identify the binding mode of PPARγ-LBD. Crystals were grown in the presence of each ligand but **6** provided only the apo structure of PPARγ. The complex with **2** provided the co-crystal structure; the crystallographic analysis data are summarized in Table S1. The overall crystal structure of the **2**/PPARγ-LBD complex was similar to that of the rosiglitazone/PPARγ-LBD complex (Figure 5). The TZD moiety formed hydrogen bonds with His323, Tyr473, Ser289, and Gln286, which was identical to that of rosiglitazone in PPARγ-LBD (Figure 6a,b). Furthermore, the coumarin moiety was positioned similar to that of the pyridine moiety in the X-ray crystal structure of the rosiglitazone/PPARγ complex (2PRG.pdb), and thus, we concluded that the transcriptional activity of probe **2** resembled that of rosiglitazone (Figure 6c,d). The N-H group on TZD of byproduct **6** was ethylated (N-Et), and therefore, it could not form a hydrogen bond with Tyr473 on helix12. Indeed, this ethyl group caused steric repulsion with helix12, and this hydrogen bond was important for PPARγ activation, explaining why probe **6** showed partial agonist activity. Importantly, although rosiglitazone is the most commonly used PPARγ ligand, no rosiglitazone-based partial agonist is reported to date. Comparison with rosiglitazone might contribute to the development of PPARγ-targeted drugs.

### 2.5. Application of PPARγ Binding Assay

**Figure 7:**
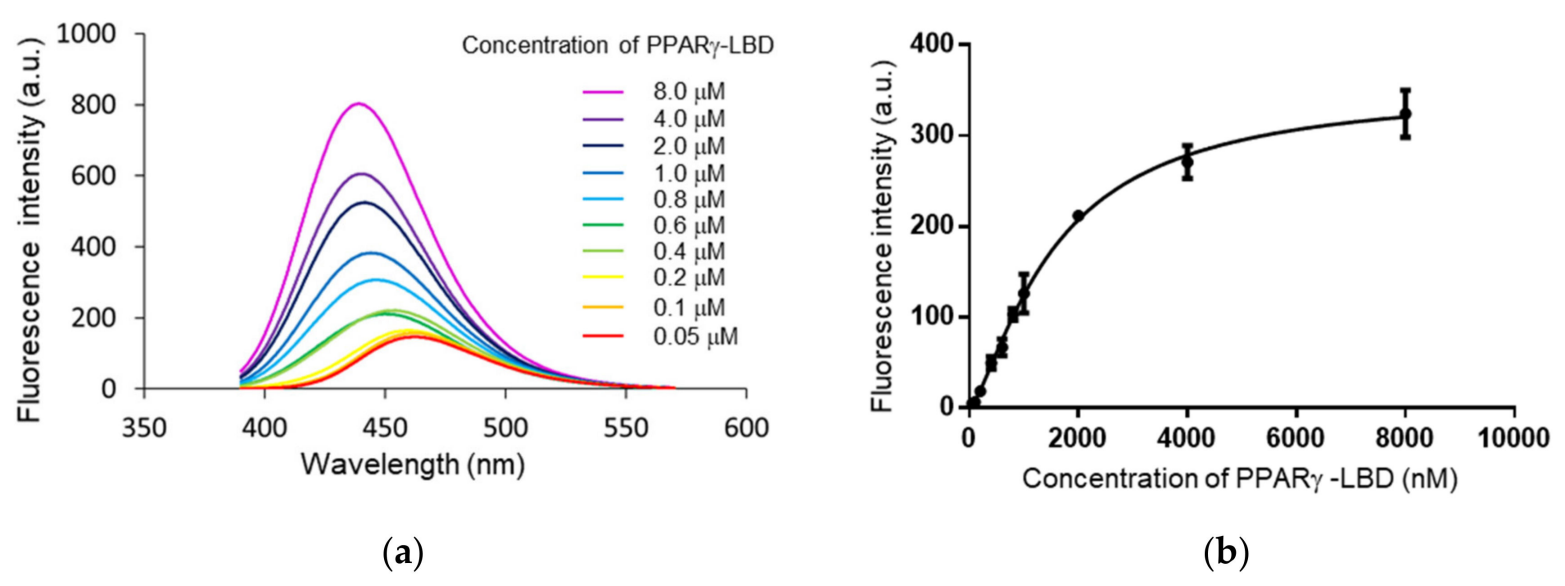
(**a**) Fluorescence spectra of **2** (1 µM) upon addition to hPPARγ-LBD (0.05–8.0 µM) in Tris–HCl buffer with Ex = 367 nm. (**b**) Fluorescence intensity of **2** at 410 nm depending on the concentration of hPPARγ-LBD. Data are mean ± SD (n = 3).

We examined whether **2** and **6** were useful for PPARγ binding assays. We attempted to determine the K_d_ value of **2** or **6** with hPPARγ-LBD. The fluorescence spectra were measured by adding hPPARγ-LBD (0.05 to 8.0 μM) in Tris–HCl buffer to **2** (1 μM) (Figure 7a). The fluorescence maxima shifted to shorter wavelength and the fluorescence intensity increased upon increasing the concentration of PPARγ-LBD. We calculated K_d_ using the fluorescence intensity at 410 nm (**2**: Kd = 1558 ± 93.61 nM, Figure 7b), (**6**: Kd = 4082 ± 712.2 nM, Figure S2). The K_d_ value showed that the PPARγ binding activity of **6** was weaker than that of **2**, and thus **6** could be useful for screening the lower affinity ligands.

**Figure 8:**
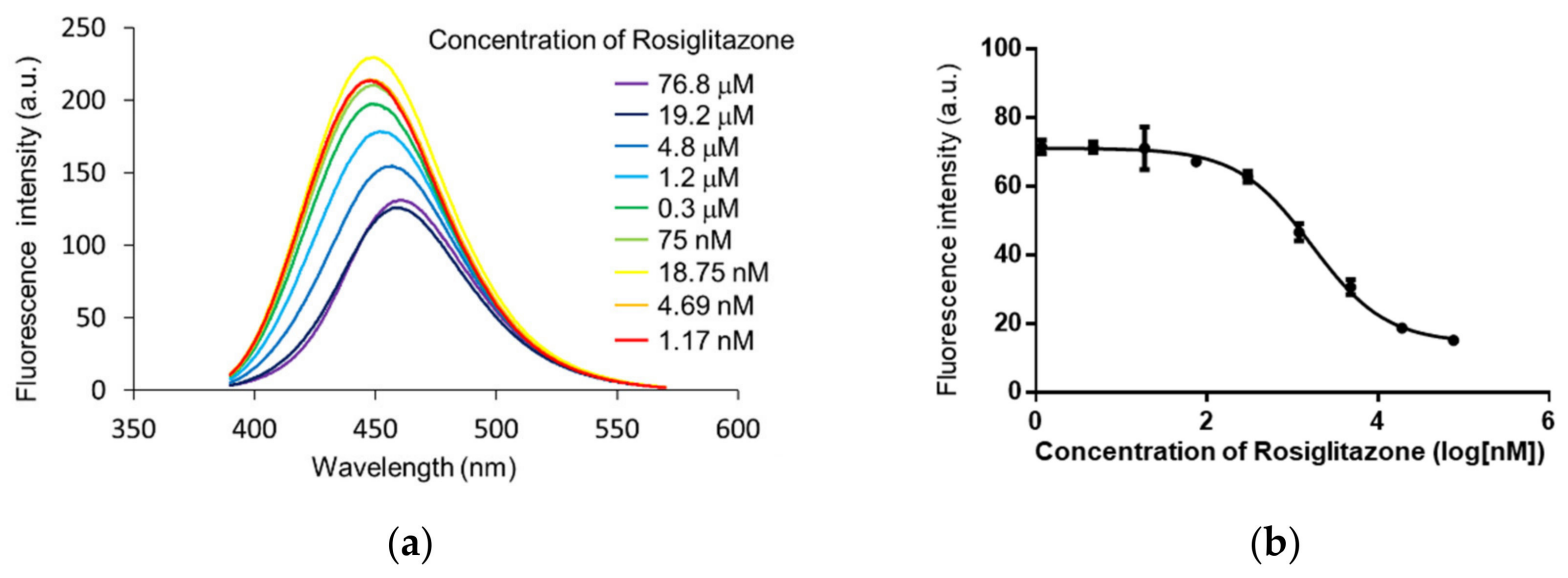
Fluorescence binding assay using the fluorescence intensity of **2** at Ex = 367 nm in the presence of 0.6 µM hPPARγ-LBD in Tris–HCl buffer. (**a**) Fluorescence spectrum of **2** (0.72 µM) and hPPARγ-LBD (0.6 µM) upon addition to rosiglitazone (1.17 nM–76.8 µM) in Tris–HCl buffer at Ex = 367 nm. (**b**) Fluorescence intensity of **2** at 410 nm in the presence of 0.6 µM hPPARγ-LBD at various concentrations of rosiglitazone. Data are mean ± SD (n = 3).

We performed PPARγ competitive binding assays using a fixed concentration of **2** (0.72 µM) and hPPARγ-LBD (0.6 µM). First, we carried out a binding assay using rosiglitazone. The addition of rosiglitazone to **2** and hPPARγ-LBD decreased the fluorescence intensity of **2** and it shifted to a longer wavelength, clearly showing that rosiglitazone replaced **2** bound to hPPARγ-LBD. We determined the value of K_i_ using the fluorescence intensity at 410 nm (K_i_ = 1157 ± 1.08 nM, Figure 8, Table 4) and that of farglitazar using the same procedure (K_i_ = 132.3 ± 1.13 nM, Figure S3, Table 4). Next, we attempted to evaluate the K_i_ value of pioglitazone, whose affinity was lower than that of rosiglitazone, but were unsuccessful because it required a concentration of pioglitazone above its solubility limit in the buffer. Therefore, we determined the K_i_ value of pioglitazone using probe **6** (1.44 µM), whose affinity was lower than that of **2**. The binding assay succeeded and we obtained the Ki value (K_i_ = 5495 ± 3.14 nM, Figure S4, Table 4). The Ki value of the PPARγ partial agonist LT175 was also determined using **6** (K_i_ = 2334 ± 1.46 nM, Figure S5, Table 4) and thus the order of the calculated K_i_ values was farglitazar < rosiglitazone < pioglitazone, consistent with the previously reported IC_50_ values for farglitazar, rosiglitazone, and pioglitazone [23] and the reported EC_50_ values [11,44] farglitazar < rosiglitazone < LT175 < pioglitazone. The determined Ki values therefore showed an identical relationship with the reported EC_50_ values, suggesting that **2** and **6**, PPARγ ligands that incorporate 7-Et_2_N coumarin, were useful probes for competitive binding assays of PPARγ ligands.

## 3. Discussion

Here, we reported the facile synthesis of PPARγ ligands incorporating coumarin, using our coumarin precursor instead of the conventional synthetic approach. We previously showed that coumarin was formed by conjugated addition from nucleophiles such as thiols, amines, alcohols, or phenols [39], and thus this strategy might be applicable to other ligands.

We also showed that **6** showed partial agonist activity caused by pushing helix12. Fewer side effects were likely caused by PPARγ partial agonists than by full agonists, such as rosiglitazone, and several partial agonists were synthesized [6] that functioned either as an “active antagonist” or a “passive antagonist”. An active antagonist regulated helix12 through direct interaction, such as steric repulsion whereas a passive antagonist interacted indirectly. Most PPARγ partial agonists act via a passive mechanism, through indirect [5] or weak [45,46] interactions or through multiple conformations [47,48]. However, probe **6** was believed to be an “active partial agonist” and pushed helix12. When we tried to co-crystallize PPARγ with **6**, we obtained only apo form crystals (data not shown). Helix12 was believed to be in an active position in the crystal packing but helix12 could not adopt its active position due to the steric repulsion of the ethyl group of **6**, resulting in the apo form that was unfavorable for crystallization. Although **6** resembled rosiglitazone, no mechanisms are reported for PPARγ partial agonism and thus probe **6** was a novel PPARγ ligand.

Competitive binding assays for nuclear receptors were either radiometric or fluorometric assays [49]. A scintillation proximity assay (SPA) was reported for PPAR [50] and an FP assay was reported for ligands containing fluorescein [22,23]. The incorporation of the 7-Et_2_N coumarin did not affect the activity of rosiglitazone, as determined by a gene transcriptional assay. We suggest that the compounds incorporating 7-Et_2_N coumarins, which are easy to synthesize, could be applied to PPARγ binding assays and did not require fluorescence anisotropy apparatus and techniques. 7-Et_2_N coumarin could be used for live cells imaging [51] and thus **2** and **6** were potent probes for cell imaging.

Recently, a coumarin-containing nuclear receptor RXR agonist was reported. The authors demonstrated its utility in a competitive binding assay for several RXR ligands [52]. Coumarins, thus, appear suitable as nuclear receptor ligands. Furthermore, it is not limited to ligands that target nuclear receptors, coumarins were reported to have an established structure for introducing fluorescence into tool compounds for the biochemical studies [53]. This was because studies on the effects of the positions of substituents and the properties of functional groups (electron-withdrawing or electron-donating) on the fluorescent properties of coumarin were widely studied for decades [54]. Moreover, some natural productss with a coumarin skeleton were reported to show PPARγ activity [55]. From this point of view, our coumarin conjugated strategy could be used to synthesize other nuclear receptor probes, photochemical probes, and bioactive compounds. Our coumarin conjugation strategy could be used to synthesize other nuclear receptor probes.

## 4. Materials and Methods

### 4.1. General Information for Synthesis

All non-aqueous reactions were performed in an oven-dried or a flame-dried glassware, under nitrogen atmosphere. Unless otherwise mentioned, all reagents were purchased from commercial suppliers and used without further purification. Organic solvents were dried by standard methods. All reactions were monitored by a thin-layer chromatography. Thin-layer chromatography was performed on silica gel 70 F_254_ TLC plates pre-coated with 0.25 mm thickness (FUJIFILM Wako Pure Chemical Corporation, Osaka, Japan). Visualization was done by UV light (254 nm or 365 nm), phosphomolybdic acid (PMA) stain, or Hanessian’s stain. Purification on silica gel column chromatography was performed on silica gel 60N (40−50 μm, 63−210 μm, Kanto Chemical Co. Inc., Tokyo, Japan). ^1^H-NMR spectra were recorded on a Bruker AV300M (300 MHz) or Bruker AV600 (600 MHz) spectrometer in appropriate deuterated solvents. ^13^C-NMR spectra were recorded at 75 MHz or 150 MHz. Chemical shifts were reported in parts per million (ppm) on the d scale from TMS peak. NMR descriptions: s, singlet; d, doublet; t, triplet; q, quartet; m, multiplet; and br, broad. Coupling constants, *J*, are reported in Hertz (Hz). High-resolution mass spectra (ESI) were obtained from a JEOL AccuTOF LC-plus JMS-T100LP spectrometer (JEOL Ltd., Tokyo, Japan).

Compounds **3** and **4** were synthesized in the same methods as in the reference [36,39]. The structures of compounds were confirmed by ^1^H-NMR.

#### 4.1.1. 5-(4-(2-((7-(diethylamino)-2-oxo-2H-chromen-4-yl) (methyl)amino)ethoxy)benzyl)thiazolidine-2,4-dione (**2**)

To a solution of **4** (19.8 mg) in DMF (0.9 mL) Et_3_N (18 μL, 0.131 mmol, 3.0 equiv.) and **3** (18.8 mg, 0.0438 mmol) in DMF (0.6 mL) was added. The mixture was stirred at 60 °C for 16 h, and then concentrated under reduced pressure. The crude solution was purified by open column chromatography (silica gel: 7 g, 5−100% AcOEt/hexane) to give **2** (12.4 mg, 0.0250 mmol, 57%, from compound **3**) and **5** (1.4 mg, 0.000485 mmol, 11%). ^1^H NMR (300 MHz, CDCl_3_) δ [ppm]: 7.63 (d, *J* = 9.0 Hz, 1H), 7.15 (d, *J* = 8.6 Hz, 2H), 6.81 (d, *J* = 8.6 Hz, 2H), 6.56−6.50 (overlapped, 2H), 5.36 (s, 1H), 4.49 (dd, *J* = 8.5, 3.9 Hz, 1H), 4.22 (m, 2H), 3.79 (m, 2H), 3.44−3.34 (overlapped, 5H), 3.18 (dd, *J* = 14.2, 8.6 Hz, 1H), 3.03 (s, 3H), 1.21 (t, *J* = 7.1 Hz, 6H). ^13^C NMR (75 MHz, CDCl_3_) δ 173.9, 170.1, 164.1, 161.6, 157.8, 156.7, 150.1, 130.7 (2 carbons), 127.9, 126.3, 114.9 (2 carbons), 107.7, 104.5, 98.2, 90.8, 65.3, 53.9, 53.4, 44.6 (2 carbons), 39.9, 37.7, 12.5 (2 carbons). ESI-HRMS: m/z calcd for C_26_H_30_N_3_O_5_S [M + H]^+^: 496.19062; found: 496.18896. IR (NaCl): 1749, 1698, 1658, 1245 cm^−1^.

#### 4.1.2. 4,7-bis(diethylamino)-2H-chromen-2-one (**5**)

^1^H NMR (300 MHz, CDCl_3_) δ [ppm]: 7.43 (d, *J* = 9.1 Hz, 1H), 6.53 (d, *J* = 9.1, 2.7 Hz, 1H), 6.48 (d, *J* = 2.6 Hz, 1H), 5.38 (s, 1H), 3.39 (quin, *J* = 7.0 Hz, 8H), 1.23 (t, *J* = 7.1 Hz, 6H), 1.20 (t, *J* = 7.1 Hz, 6H). ^13^C NMR (75 MHz, CDCl_3_) δ [ppm]: 164.0, 160.1, 156.7, 149.9, 126.1, 107.5, 105.1, 98.2, 90.7, 45.5 (2 carbons), 44.6 (2 carbons), 12.5 (2 carbons), 12.3 (2 carbons). ESI-HRMS: m/z calcd for C_17_H_24_N_2_O_2_ [M + H]^+^: 289.19160; found: 289.19162. IR (NaCl): 1698 cm^−1^.

#### 4.1.3. 5-(4-(2-((7-(diethylamino)-2-oxo-2H-chromen-4-yl)(methyl)amino)ethoxy)benzyl)-3-ethylthiazolidine-2,4-dione (**6**)

^1^H NMR (300 MHz, CDCl_3_) δ [ppm]: 7.61 (d, *J* = 9.0 Hz, 1H), 7.17−7.12 (overlapped, 2H), 6.86−6.81 (overlapped, 2H), 6.55−6.49 (overlapped, 2H), 5.43 (s, 1H), 4.41 (dd, *J* = 9.0, 3.9 Hz, 1H), 4.21 (t, *J* = 5.6 Hz, 2H), 3.76 (t, *J* = 5.5 Hz, 2H), 3.61 (m, 2H), 3.46−3.35 (overlapped, 5H), 3.09 (dd, *J* = 14.1, 8.9 Hz, 1H), 3.04 (s, 3H), 1.20 (t, *J* = 7.1 Hz, 6H), 1.11 (t, *J* = 7.2 Hz, 3H). ^13^C NMR (75 MHz, CDCl_3_) δ [ppm]: 173.8, 170.9, 163.7, 161.5, 157.8, 156.7, 150.1, 130.6 (2 carbons), 128.3, 126.3, 114.7 (2 carbons), 107.6, 104.5, 98.2, 91.0, 65.3, 53.7, 51.6, 44.6 (2 carbons), 39.9, 37.8, 36.9, 12.8, 12.5 (2 carbons). ESI-HRMS: m/z calcd for C_28_H_34_N_3_O_5_S [M + H]^+^: 524.22192; found: 524.22030. IR (NaCl): 1745, 1682, 1613 cm^−1^.

### 4.2. General Information for Biological Experiments

All biological reagents were used without further purification, unless otherwise noted. Sodium dodecyl sulfate-polyacrylamide gel electrophoresis (SDS-PAGE) was carried out with an AE-6530 electrophoresis apparatus. UV-visible absorption spectra were recorded on a V-630BIO spectrophotometer (JASCO, Tokyo, Japan). Fluorescent spectra were measured using a F-7100 fluorescence spectrophotometer (HITACHI, Tokyo, Japan).

### 4.3. Transactivation Assay

Transactivation in COS-7 cells was measured using a dual luciferase assay according to a previously reported procedure [46]. EC_50_s were calculated by GraphPad Prism 6 (GraphPad Software, San Diego, USA) (<A>LogEC = LogEC_50_Control <~A>LogEC = LogEC_50_Control + log(EC_50_Ratio) Y = Bottom + (Top-Bottom)/(1 + 10^((LogEC-X) × HillSlope))).

### 4.4. Protein Expression and Purifications

PPARγ expression and purification were carried out as previously described [46]. The human PPARγ-LBD (residues 204−477) was expressed using a modified pET30a vector with an N-terminal 6×His tag cleavable by TEV protease. *E. coli* Rosetta (DE3) was freshly transformed with the plasmid and grown 1 L of 2 × TY medium with 34 mg/mL kanamycin and 50 mg/mL chloramphenicol at 37 °C, to an OD at 600 nm of 0.6−1.0. Protein synthesis was then induced with 0.5 mM isopropyl-b-Dthiogalactopyranoside (IPTG), and the cultures were further incubated at 20 °C for 16 h. Cells were harvested and resuspended in 50 mL of lysis buffer (20 mM Tris–HCl pH 8.0, 100 mM NaCl, 1 mM TCEP, 0.5 mM EDTA, and 13 Protease inhibitor cocktail). Cells were lysed by sonication, and the soluble fraction was isolated by centrifugation (18,000× *g* for 30 min). The supernatant was applied to cOmplete His-Tag Purification Resin (Roche, Basel, Switzerland), and the resin was thoroughly washed in wash buffer (20 mM Tris–HCl pH 8.0, 100 mM NaCl, 1 mM TCEP, and 5 mM imidazole). The human PPARγ-LBD was eluted with the elution buffer (20 mM Tris–HCl pH 8.0, 100 mM NaCl, 1 mM TCEP, and 250 mM imidazole). TEV protease was added to the eluate, and the mixture was dialyzed overnight at 4 °C with 500 mL of buffer (20 mM Tris–HCl pH 8.0, 1 mM TCEP, 0.5 mM EDTA). The cleaved protein was passed through complete His-Tag Purification Resin (Roche). The flow-through was loaded onto a Resource Q (6 mL) column (GE Healthcare, Chicago, USA) equilibrated with buffer (20 mM Tris–HCl pH 8.0, 1 mM TCEP, 0.5 mM EDTA). The column was eluted with an NaCl gradient from 0 to 1 M in the starting buffer. The eluted fractions were concentrated and loaded onto a Superdex 75 Increase 10/300 GL gel filtration (24 mL) column (GE Healthcare) equilibrated with buffer (20 mM Tris–HCl pH 8.0, 1 mM TCEP, and 0.5 mM EDTA). Purified human PPARγ-LBD was concentrated in buffer (20 mM Tris–HCl pH 8.0, 1 mM TCEP, and 0.5 mM EDTA) to 6.0 mg/mL, which was estimated by UV absorbance at 280 nm.

### 4.5. X-Ray Crystallography

Crystals were obtained through co-crystallization in ligand **2**. For the PPARγ, co-crystallization was performed by vapor diffusion using a hanging drop made by mixing 1 μL of the PPARγ-LBD solution (6 mg/mL, in 20 mM Tris–HCl pH 8.0, 1 mM TCEP, 0.5 mM EDTA) with 0.5 equivalent Ligand, (**2**) with 1 μL of reservoir solution (0.8 M sodium citrate and 0.1 M Tris–HCl pH 7.27) and the drops were equilibrated against 300 μL of reservoir solution at room temperature. The mixture was stored at room temperature, and crystals appeared after about 2 weeks. Crystals were flash-cooled in liquid nitrogen, after a fast soaking in a cryoprotectant buffer (LV Cryo Oil (MiTeGen, NY, USA)). Diffraction data sets were collected at 100 K in a stream of nitrogen gas at beamline BL-5A, at the high energy accelerator research organization (KEK, Tsukuba, Japan). Reflections were recorded with an oscillation range per image of 1.0°. Diffraction data were indexed, integrated, and scaled using the program iMOSFLM (MRC-LMB, Cambridge, UK) [56,57]. The ternary complex structures were solved by molecular replacement with the software Phaser [58] in the CCP4 program (Research Complex at Harwell, Oxford, UK) [59] using rat VDR-LBD coordinates (PDB code: 2VV3) [7], and the finalized sets of atomic coordinates were obtained after iterative rounds of model modification with the program Coot (MRC-LMB, Cambridge, UK) [60] and refinement with refmac5 (University of York, York, UK) [61,62,63,64,65].

### 4.6. UV-Visible Absorption and Fluorescence Spectroscopic Analyses

Stock solutions of model coumarin compounds PPAR ligands **1**, **2**, and **6** were prepared in DMSO, and stored in the dark at −20 °C. The stock solutions were diluted (2 μM) with solvents (CH_2_Cl_2_, THF, MeOH, and H_2_O) and then the UV-visible absorption and fluorescence signals were measured by a spectrometer. The fluorescence quantum yields of coumarin analogues were calculated using quinine sulfate (Φ = 0.577 in 0.1 M H_2_SO_4_) as a reference standard [43].

### 4.7. K_d_ Determination of **2** or **6**

PPARγ-LBD (6 mg/mL) was diluted to the concentration of 0.05, 0.1, 0.2, 0.4, 0.6, 0.8, 1.0, 2.0, 4.0, and 8.0 μM (0.25, 0.5, 1.0, 1.5, 2.0, 3.0, 4.0, 5.0, 10.0, and 15.0 μM, in case of **6**) with the assay buffer (20 mM Tris–HCl pH 7.0, 1 mM TCEP, and 0.5 mM EDTA). Then, each concentration levels of PPARγ-LBD solutions were added **2** or **6** (final concentration of 1 μM), and the fluorescence spectra were measured using the mixture of PPARγ-LBD and **2**, or **6** (200 μL), using a quartz cuvette (5 mm). The assay buffer was measured as the background for fluorescence spectrum. The excitation wavelength of fluorescence spectra was set at 367 nm, and emission was detected from 350 nm to 570 nm. The specific equilibrium binding constant (K_d_) was derived using GraphPad Prism6 (Y = B_max_ × X^h/(K_d_^h + X^h) (X: concentration of PPARγ-LBD [nM], Y: fluorescence intensity at 410 nm, h: Hill slope).

### 4.8. Binding Assay of Rosiglitazone or Farglitazar with hPPARγ- LBD Using **2**

PPARγ-LBD (6 mg/mL) was diluted to the concentration of 0.6 μM with the assay buffer, and PPARγ-LBD solution was added **2** (0.72 μM final concentration). Then, four-fold serial dilutions of Rosiglitazone or Farglitazar (Rosiglitazone: final concentration of 1.17 nM to 76.8 μM, 9 concentration levels, Farglitazar: final concentration of 0.59 nM to 9.6 μM, 8 concentration levels) was added to the mixture, and the fluorescence spectra were measured using the mixture of PPARγ-LBD, **2**, and Rosiglitazone or Farglitazar (200 μL), using a quartz cuvette (5 mm). The assay buffer was measured as the background for the fluorescence spectrum. The excitation wavelength of fluorescence spectra was set at 367 nm, and emission was detected from 350 nm to 570 nm. The inhibition constant (K_i_) value was derived from K_d_ of **2**, using GraphPad Prism6 (logEC_50_ = log(10^logK_i_ × (1 + Radioligand [nM]/HotK_d_ [nM])) Y = Bottom + (Top-Bottom)/(1 + 10^(X-LogEC_50_))) (X: concentration of Rosiglitazone or Farglitazar [nM], Y: fluorescence intensity at 410 nm, Radioligand [nM]: concentration of **2**, HotK_d_ [nM]: the K_d_ value of **2**).

### 4.9. Binding Assay of Pioglitazone or LT175 with hPPARγ- LBD Using **6**

PPARγ-LBD (6 mg/mL) was diluted to the concentration of 0.6 μM with the assay buffer, and PPARγ-LBD solution was added **6** (1.44 μM final concentration). Then, four-fold serial dilutions Pioglitazone or LT175 (final concentration of 4.69 nM to 76.8 μM, 8 concentration levels) was added to the mixture. Next, the fluorescence spectra were measured using the mixture of PPARγ-LBD, **6**, and Pioglitazone or LT175 (200 μL) using a quartz cuvette (5 mm). The assay buffer was measured as the background for fluorescence spectrum. The excitation wavelength of fluorescence spectra was set at 367 nm, and emission was detected from 350 nm to 570 nm. The inhibition constant (K_i_) value was derived from K_d_ of **6** using GraphPad Prism6 (logEC_50_ = log(10^logK_i_ × (1 + Radioligand [nM]/HotK_d_ [nM])) Y = Bottom + (Top-Bottom)/(1 + 10^(X-LogEC_50_))) (X: concentration of Pioglitazone or LT175, Y: fluorescence intensity at 410 nm, Radioligand [nM]: concentration of **6**, HotK_d_ [nM]: the K_d_ value of **6**).

## 5. Conclusions

To efficiently evaluate the ligands for PPARγ, a target molecule for metabolic syndrome and inflammatory diseases, we synthesized compound **2** using our method. In the process, we also obtained partial agonist **6**, which appeared to act via a novel mechanism. By utilizing **2** and **6**, we showed that a fluorescence spectrophotometer could be used to evaluate PPAR**γ** binding affinity. These results might contribute to the understanding of metabolic syndrome and inflammation, as well as drug development.

## Figures and Tables

**Table 1:** Investigation of PPARγ ligand synthesis conditions.

Entry	Solvent	Base	Temperature (°C)	Yield (%)		
				2	5	6
1	Et_3_N	-	100	-	40	-
2	DMF: Et_3_N = 1:1	-	100	14	51	14
3	DMF	Et_3_N (3.0 equiv.)	60	57	11	-

**Table 2:** The EC_50_ values of compounds **1**, **2**, and **6**.

Compound	Rosiglitazone	1	2	6
EC_50_ (μM)	0.17	0.18	0.034	0.35

**Table 3:** Fluorescence properties for PPARγ ligands **1**, **2**, and **6**.

Comp.	In CH_2_Cl_2_		In THF		In MeOH		In H_2_O	
	Abs_max_^1^ Em_max_ Δ ^3^ (nm)	Q.Y. ^2^	Abs_max_^1^ Em_max_ Δ ^3^ (nm)	Q.Y. ^2^	Abs_max_^1^ Em_ma_ Δ ^3^ (nm)	Q.Y. ^2^	Abs_max_^1^ Em_max_ Δ ^3^ (nm)	Q.Y. ^2^
1	316 469 153	0.00359	314 467 153	0.00461	314 392 78	0.0039	312 451 139	0.00369
2	359 423 64	0.541	354 416 62	0.191	359 449 90	0.532	364 462 98	0.143
6	359 423 64	0.764	353 416 63	0.172	358 449 91	0.432	369 461 92	0.0865

^1^ See the Supplementary Materials (Figure S1) for absorption spectrum of **2** and **6**. ^2^ The quantum yields were determined using quinine sulfate in 0.1 M H_2_SO_4_ (Φ = 0.577) [43]. ^3^ **Δ** = Abs_max_ − Em_max_.

**Table 4:** Comparison of the K_i_ value determined using **2** or **6** with reported IC_50_ or EC_50_ values.

Concentration (nM) [ref]	Rosiglitazone	Farglitazar	Pioglitazone	LT175
The calculated K_i_ value using **2** or **6** ^1^	1157 ± 1.08	132.3 ± 1.13	5495 ± 3.14	2334 ± 1.46
The reported IC_50_ value [23]	660 ± 252	90 ± 34	4500 ± 465	-
The reported EC_50_ value [44]	18 ± 4	0.20 ± 0.05	280 ± 42	-
The reported EC_50_ value [11]	40 ± 20	-	-	480 ± 80

^1^ Data are mean ± SD (n = 3).

## Data Availability

Data are contained within the article or Supplementary Materials.

## Supplementary Materials

The following are available online at https://www.mdpi.com/article/10.3390/ijms22084034/s1. Scheme S1: The proposed mechanism of reaction for synthesis of **5**. Scheme S2: The proposed mechanism of reaction for synthesis of **6**. Figure S1: Absorption spectrum of **2** and **6**. Figure S2: Kd determination of 6. Figure S3: Binding assay of Farglitazar for hPPARγ—LBD using **2**. Figure S4: Binding assay of Pioglitazone for hPPAR—LBD using **6**. Figure S5: Binding assay of LT175 for hPPARγ—LBD using **6**. Spectra of compounds **2**, **5**, and **6**(^1^H NMR, ^13^C NMR). Table S1: Data collection and refinement statistics of the crystal structures.
