# Supplementary material for: Synthesis of a Coumarin-Based PPARγ Fluorescence Probe for Competitive Binding Assay
Source: Int J Mol Sci. 2021 Apr 14;22(8):4034. doi: 10.3390/ijms22084034 (PMC8070791; doi:10.3390/ijms22084034)
Supplement: Supplementary file 1 [file ijms-22-04034-s001.pdf]

Synthesis of a Coumarin Based PPARγ Fluorescence Probe for Competitive Binding Assay

Chisato Yoshikawa, Hiroaki Ishida, Nami Ohashi, Toshimasa Itoh*

Laboratory of Drug Design and Medicinal Chemistry, Showa Pharmaceutical University, 3-3165 Higashi- tamagawagakuen, Machida, Tokyo 194-8543, Japan
**Corresponding author: email: titoh@ac.shoyaku.ac.jp (T. Itoh)*

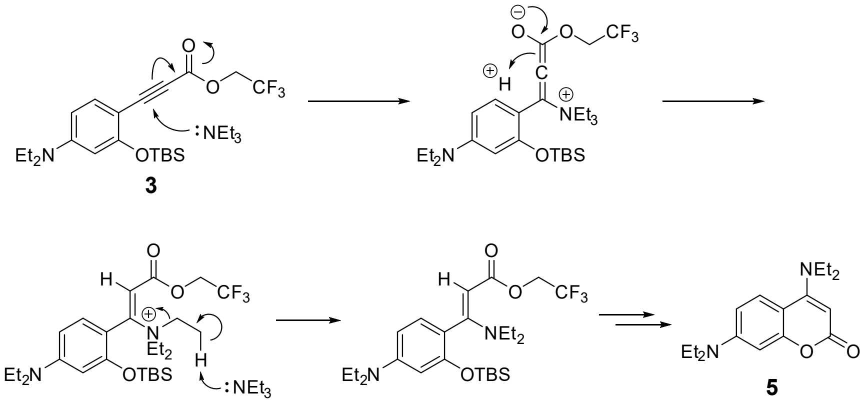

Scheme S1. The proposed mechanism for accessing **5**.

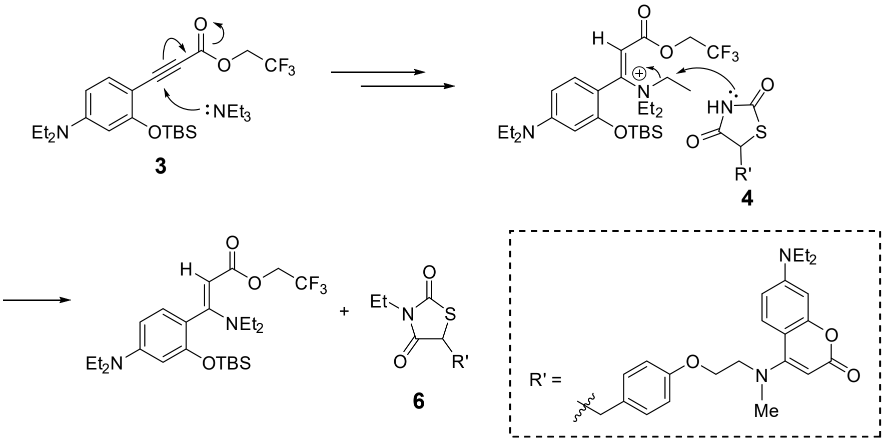

Scheme S2. The proposed mechanism for accessing **6**.

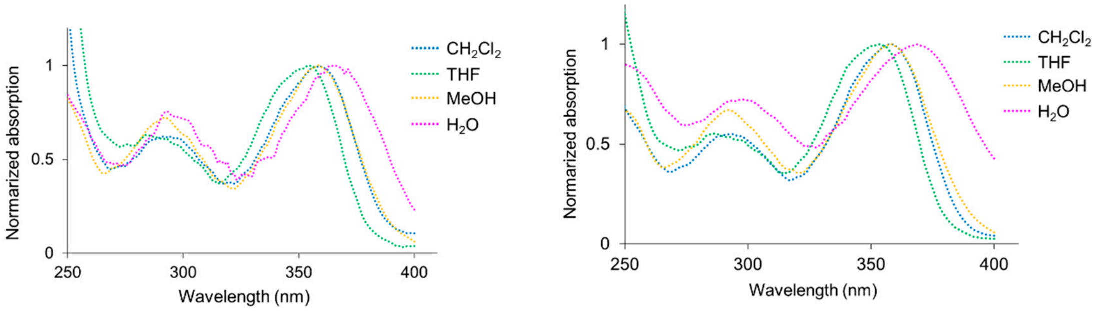

(**a**) (**b**)

Figure S1. (**a**) Absorption spectrum of **2**, in CH_2Cl_2, THF, MeOH, or H_2O (2μM). (**b**) Absorption spectrum of **6**, in CH_2Cl_2, THF, MeOH, or H_2O (2μM).

Table S1. Data collection and refinement statistics of the crystal structures.

**Data collection**			
Space group	C 1 2 1		
Cell dimensions			
a, *b*, *c* (Å)	93.33, 61.9, 119.37		
α, β, γ (°)	90, 102.29, 90		
Resolution (Å)	Overall	Inner	Outer
Low resolution limit	26.75	26.75	2.38
High resolution limit	2.30	8.91	2.30
R_{merge}	0.051	0.021	0.400
Total number of reflections	87204	1830	8301
No. of unique reflections	29512	533	2829
completeness (%)	99.1	96.2	97.0
Redundancy	3.0	3.4	2.9

**Refinement**	
Resolution range (Å)	26.75-2.30
No. reflections all/free	29502/1515
R-factor/R-free	0.224 / 0.264
RMS Deviations	
Bond lengths (Å)	0.014
Bond angles (°)	1.886
No. atoms	
Protein	8150
Ligand/ion	64
Water	72
B-factors	
Protein	32.1
Ligand/ion	37.8
Water	48

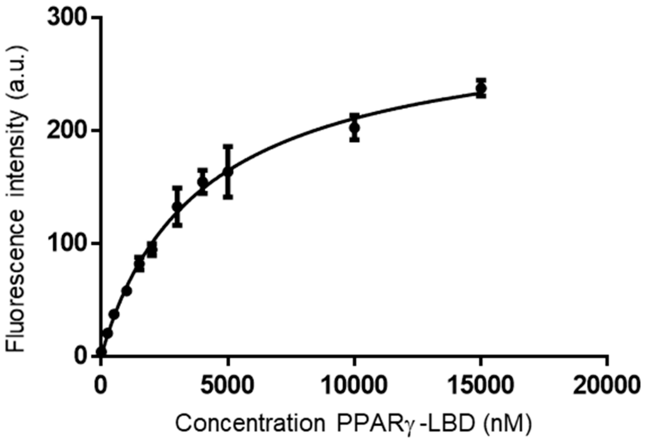

Figure S2. K_d determination of **6**. Fluorescence intensity of **6** at 410 nm depending on the concentration of hPPARγ-LBD. Data are mean ± SD (n = 3).

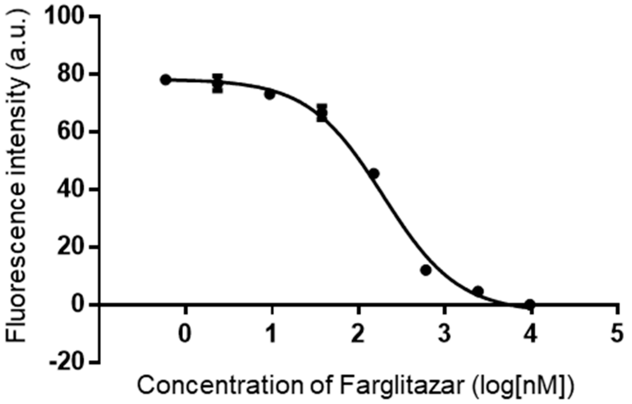

Figure S3. Binding assay of Farglitazar for hPPARγ- LBD using **2**. Fluorescence intensity of **2** (0.72 μM) at 410 nm in the presence of 0.6 μM hPPARγ-LBD depending on the concentration of Farglitazar (0.59 nM–9.6 μM). Data are mean ± SD (n = 3).

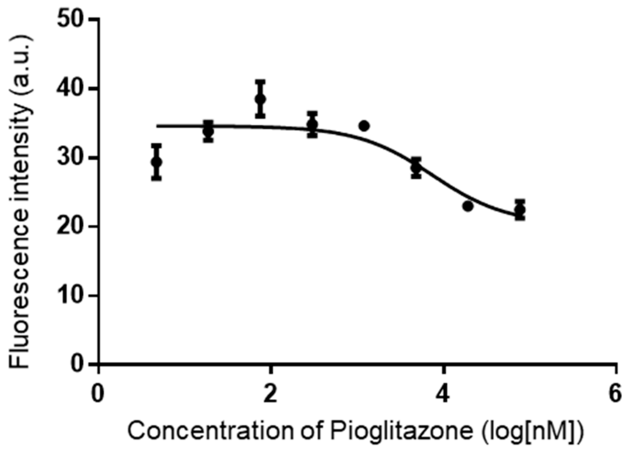

Figure S4. Binding assay of Pioglitazone for hPPARγ- LBD using **6**. Fluorescence intensity of **6** (1.44 μM) at 410 nm in the presence of 0.6 μM hPPARγ-LBD depending on the concentration of Pioglitazone (4.69 nM–76.8 μM). Data are mean ± SD (n = 3).

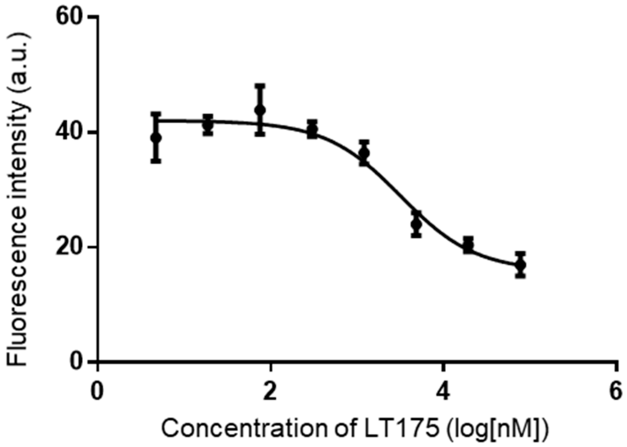

Figure S5. Binding assay of LT175 for hPPARγ- LBD using **6**. Fluorescence intensity of **6** (1.44 μM) at 410 nm in the presence of 0.6 μM hPPARγ-LBD depending on the concentration of LT175 (4.69 nM–76.8 μM). Data are mean ± SD (n = 3).

Spectra of compounds (^{1}H NMR, ^{13}C NMR)

^{1}H NMR spectrum of compound 2 in $CDCl_3$ (300 MHz)

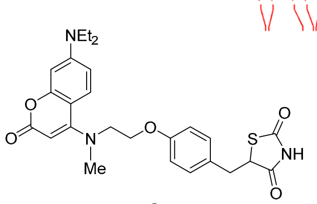

2

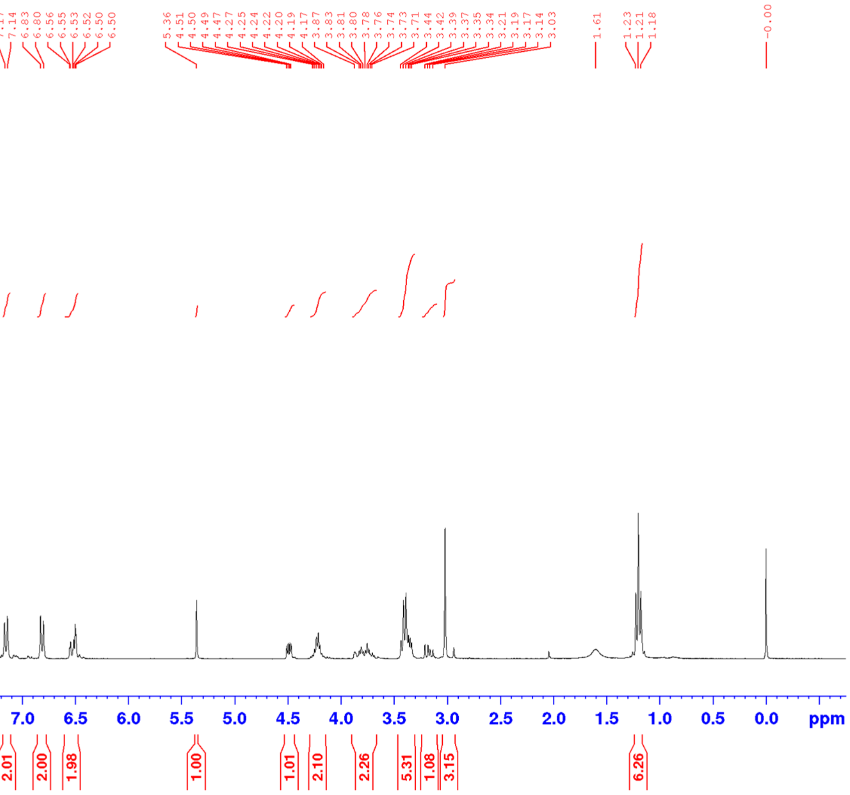

^{13}C NMR spectrum of compound 2 in $CDCl_3$ (75 MHz)

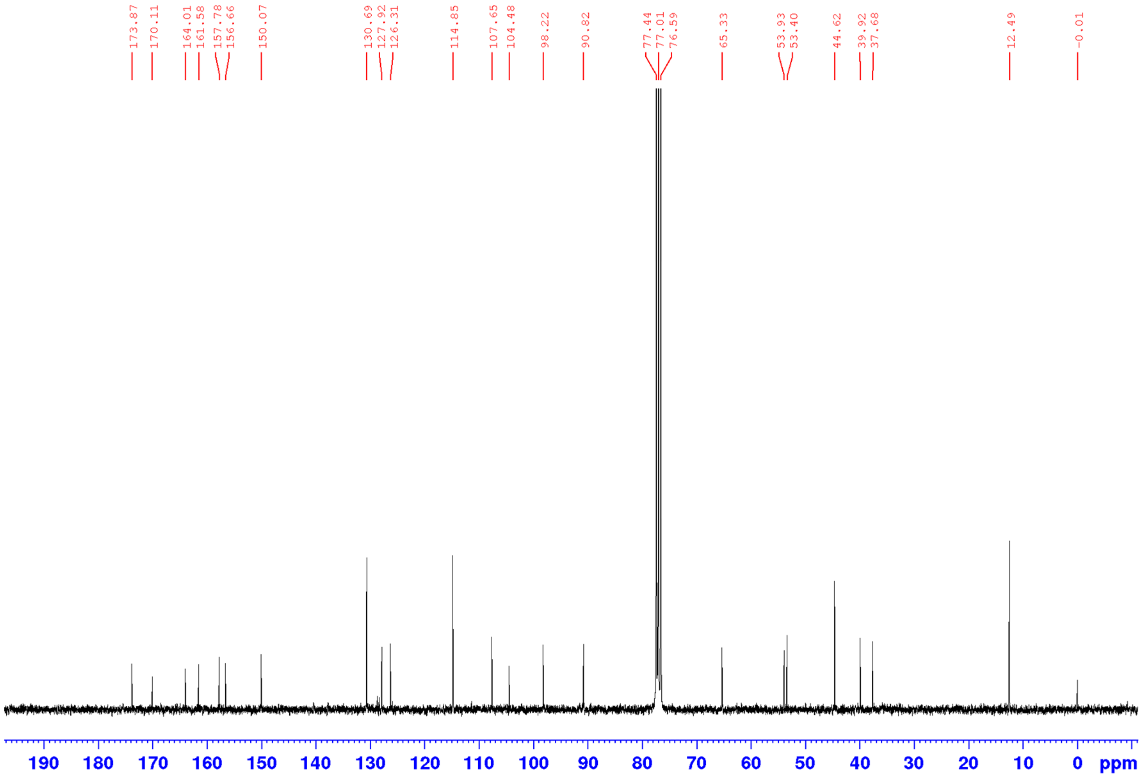

^{1}H NMR spectrum of compound 5 in $CDCl_3$ (300 MHz)

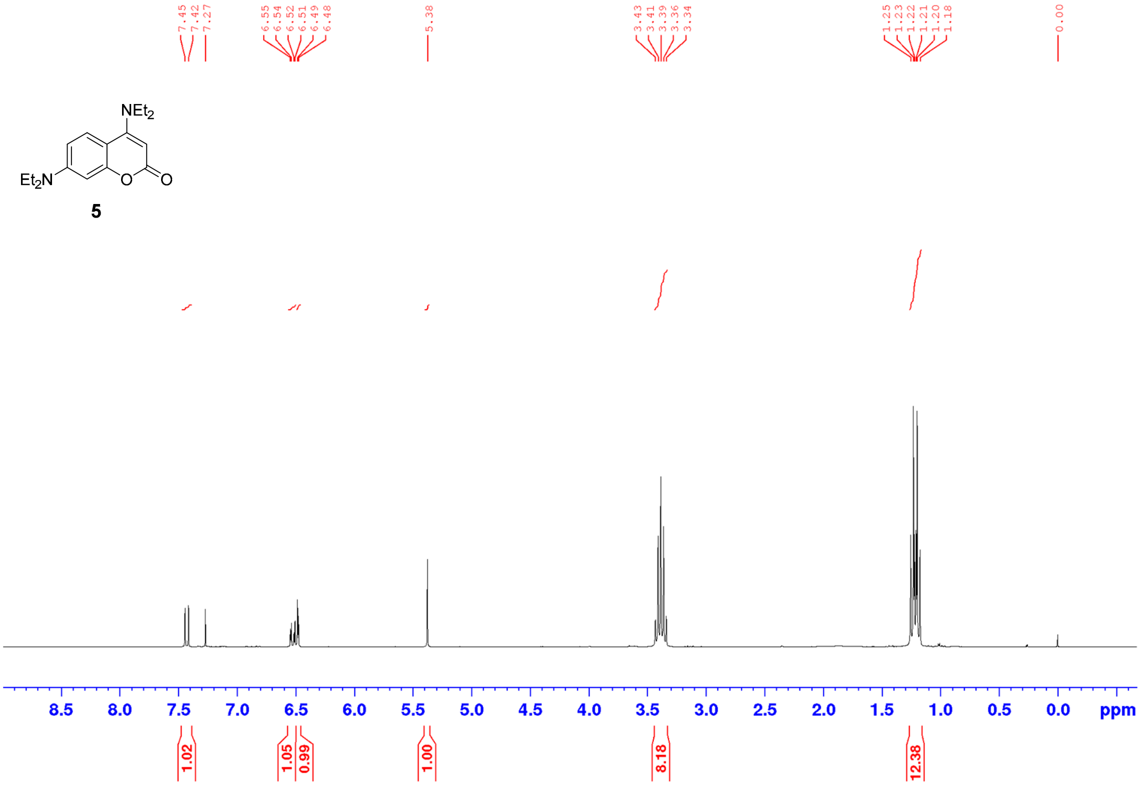

^{13}C NMR spectrum of compound 5 in $CDCl_3$ (75 MHz)

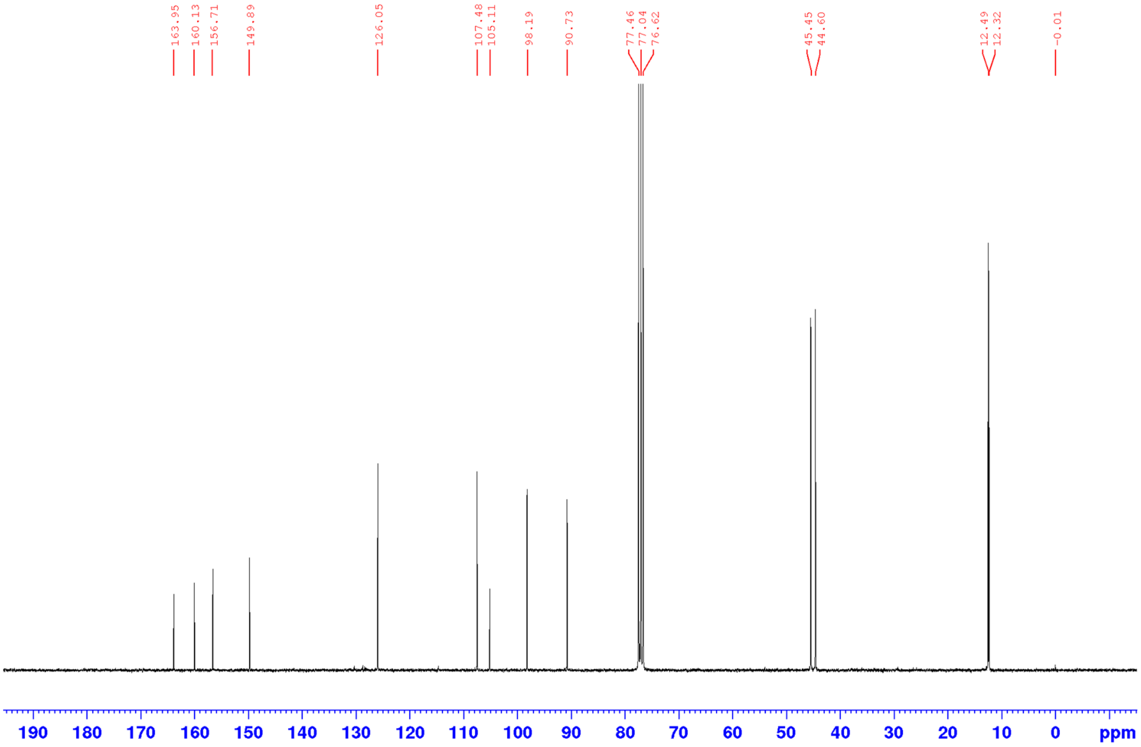

^{1}H NMR spectrum of compound 6 in $CDCl_3$ (300 MHz)

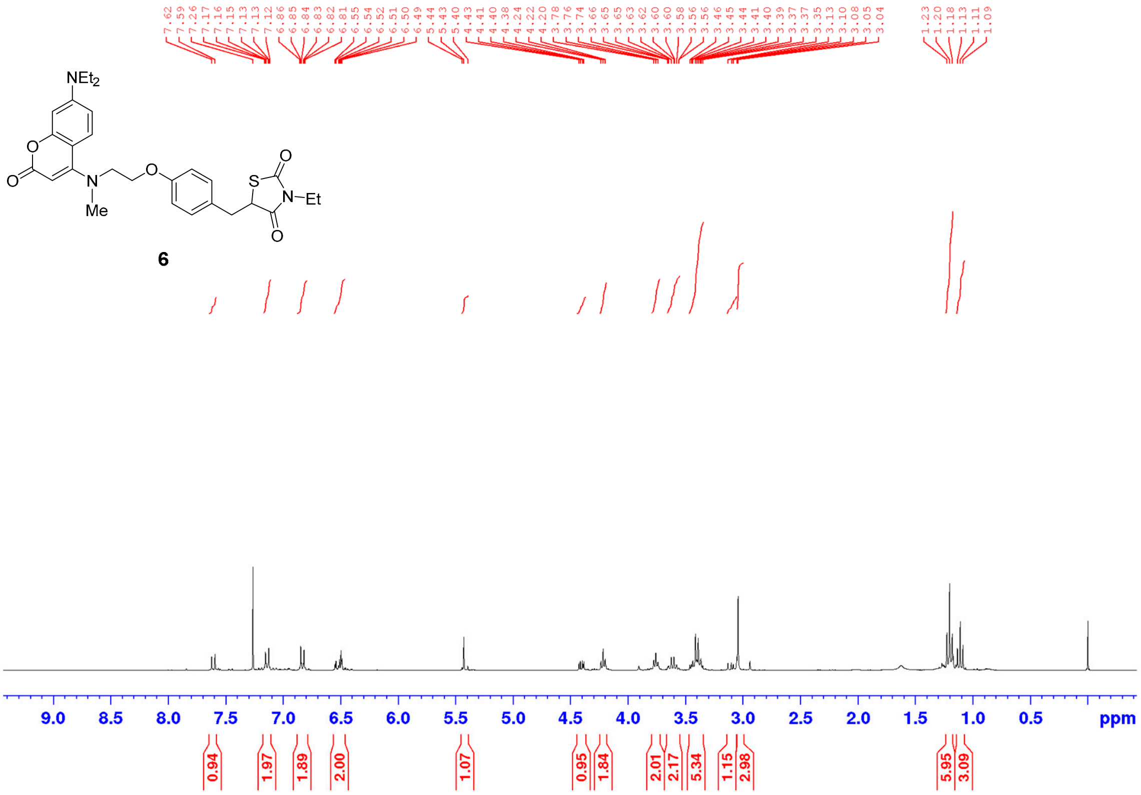

^{13}C NMR spectrum of compound 6 in $CDCl_3$ (75 MHz)

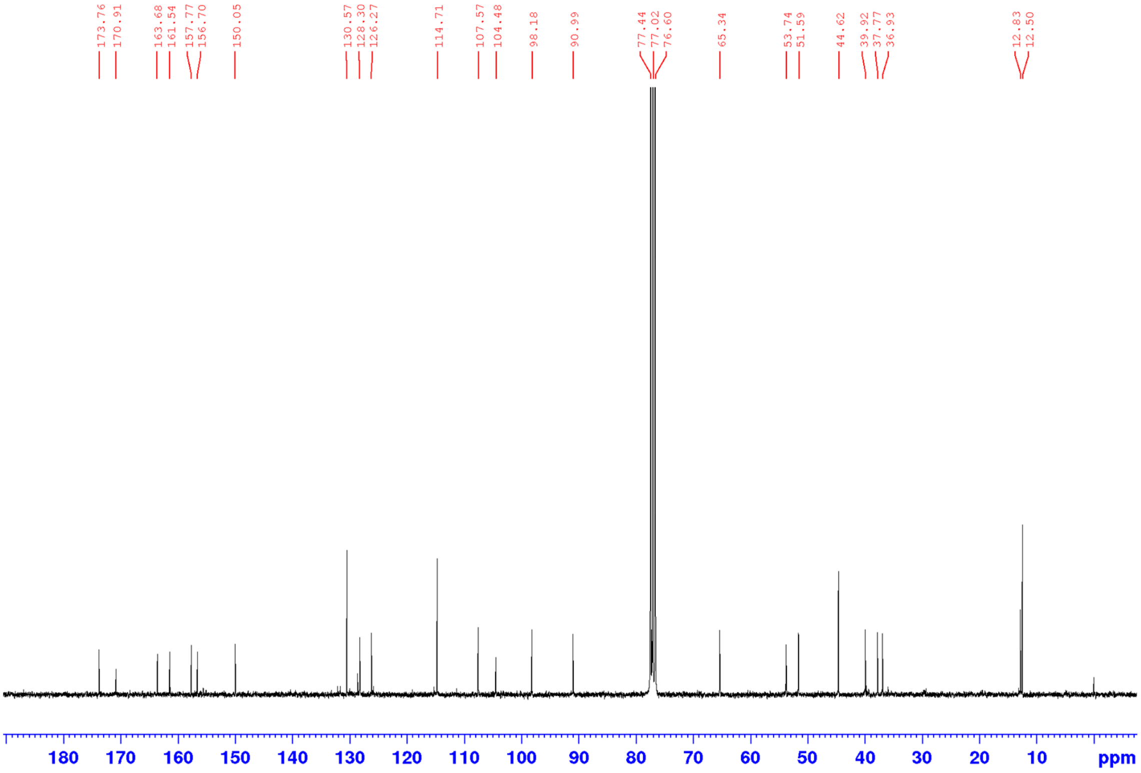

Biological experiments section

Cell Lines

COS-7 cells (RIKEN BRC) was cultured in Dulbecco's Modified Eagle's medium (DMEM) (Sigma-Aldrich) supplemented with 5% heat inactivated fetal bovine serum (FBS) (HyClone) and 1% penicillin-streptomycin (containing 10000 U/mL penicillin and 10000 μg/mL streptomycin; Nacalai Tesque).
